# RNA splicing factor USP39 promotes glioma progression by inducing TAZ mRNA maturation

**DOI:** 10.1038/s41388-019-0888-1

**Published:** 2019-07-22

**Authors:** Kaikai Ding, Jianxiong Ji, Xin Zhang, Bin Huang, Anjing Chen, Di Zhang, Xingang Li, Xinyu Wang, Jian Wang

**Affiliations:** 1 Department of Neurosurgery, Qilu Hospital of Shandong University and Institute of Brain and Brain-Inspired Science, Shandong University, 250012 Jinan, PR China; 2 Shandong Key Laboratory of Brain Function Remodeling, 250012 Jinan, PR China; 3 Department of Biomedicine, University of Bergen, 5009 Bergen, Norway

**Keywords:** Oncogenes, Mechanisms of disease

## Abstract

Increasing evidence demonstrates that ubiquitin specific protease 39 (USP39) plays an oncogenic role in various human tumors. Here, using expression analysis of the publicly available Oncomine database, clinical glioma patient samples, and glioma cells, we found that USP39 was overexpressed in human gliomas. Knockdown of USP39 in glioma cells demonstrated that the protein promoted cell growth, invasion and migration in vitro and in a tumor model in nude mice. To identify mediators of USP39 growth-promoting properties, we used luciferase reporter constructs under transcriptional control of various promoters specific to seven canonical cancer-associated pathways. Luciferase activity from a synthetic TEAD-dependent YAP/TAZ-responsive reporter, as a direct readout of the Hippo signaling pathway, was decreased by 92% in cells with USP39 knockdown, whereas the luciferase activities from the other six cancer pathways, including MAPK/ERK, MAPK/JNK, NFκB, Notch, TGFβ, and Wnt, remained unchanged. TAZ protein expression however was decreased independent of canonical Hippo signaling. Immunohistochemistry revealed a positive correlation between USP39 and TAZ proteins in orthotopic xenografts derived from modified glioma cells expressing USP39 shRNAs and primary human glioma samples (*p* < 0.05). Finally, loss of USP39 decreased *TAZ* pre-mRNA splicing efficiency in glioma cells in vitro, which led to reduced levels of TAZ protein. In summary, USP39 has oncogenic properties that increase TAZ protein levels by inducing maturation of its mRNA. USP39 therefore provides a novel therapeutic target for the treatment of human glioma.

## Introduction

Malignant gliomas are the most common aggressive and fatal primary brain tumors in adults [1, 2]. Despite great advances in treatment, median survival for patients with glioblastoma (GBM), the most aggressive glioma, is 14.5–16.6 months [3]. The current standard of care is largely ineffective due to complex features, which include aggressive growth and diffuse invasion. In recent years, much of the research effort has focused on the molecular basis of the disease with the goal of translating this better understanding into viable therapies [4].

Ubiquitin specific protease 39 (USP39) is a member of a family of deubiquitinating proteins that are classified based on the sequence similarity in the Dub-domain. However, USP39 is interestingly entirely deprived of deubiquitinating activity due to the absence of conserved active-site residues (a cysteine, histidine, and aspartic acids) in the Dub-domain [5]. USP39 is also known as Sad1p in yeast and a 65 kDa SR-related protein in humans, both of which have been implicated in assembly of the mature spliceosome complex, suggesting a role in mRNA splicing [6–8]. Previous studies have demonstrated that USP39 is involved in splicing of the transcript for Aurora B [5]. Mutation of zebrafish USP39 induces mRNA splicing defects in the retinoblastoma gene, rb1 [9]. USP39 is also reported to be a pivotal regulator of *EGFR* pre-mRNA splicing [10]. In addition, an oncogenic role for USP39 has been reported in many malignant tumors, such as prostate cancer [10], oral squamous cell carcinoma [11], gastric cancer [12], melanoma [13], osteosarcoma [14], breast cancer [15], hepatocellular carcinoma [16], medullary thyroid carcinoma [17], lung cancer [18], pancreatic cancer [19], colorectal cancer [20], and renal cell carcinoma [21]. However, the role of USP39 in glioma has not been well defined.

TAZ, also known as WWTR1 (WW-domain containing transcriptional regulator 1), a transcriptional coactivator with a PDZ-binding motif, and its paralog, yes-associated protein (YAP), are associated primarily with activity in the Hippo tumor suppressor pathway, which plays a prominent role in cell proliferation, cell apoptosis, tumor metastasis, and maintenance of stem cell traits in cancer [22–25]. Classic Hippo signaling is transmitted through a kinase cascade. The upstream kinases, MST1 and MST2, work together with the adaptor protein SAV1/WW45 to phosphorylate and activate LATS1 and LATS2. These activated LATS kinases together with MOB1 then phosphorylate the TAZ and YAP effector proteins [26]. Phosphorylated TAZ is mainly localized in the cytoplasm, whereas unphosphorylated TAZ enters the nucleus, where the protein functions as a transcriptional coactivator to promote tumor growth through the induction of gene transcription [27–29]. Elevated expression and activity of TAZ have been identified in various human cancers, such as melanoma, hepatocellular carcinoma, and breast, liver, and colorectal cancers [24, 30–32]. In addition, TAZ expression has been found to be increased, positively correlated with tumor grade, and involved in regulating mesenchymal differentiation in glioma [33, 34]. However, several aspects of the role of TAZ in glioma remain unknown.

In this work, we investigate the expression of USP39 in human gliomas and comprehensively study the functional role of USP39 in the development of the disease using model systems both in vitro and in vivo. We reveal that USP39 has growth-promoting properties in human gliomas, and investigation of putative oncogenic mechanisms illuminates a role in mRNA processing of TAZ, a transcriptional regulator with known oncogenic properties. USP39 may thus be a potential prognostic biomarker and therapeutic target in the treatment of glioma patients.

## Results

### Increased expression of USP39 is associated with increasing tumor grade in primary human gliomas

We first analyzed *USP39* expression in human cancer in the publicly available database Oncomine. Results demonstrated that *USP39* was upregulated in many tumor types, including brain and CNS cancer (Supplementary Fig. 1a). We then analyzed *USP39* mRNA expression specifically in GBM, and found that the numbers of samples in TCGA and Sun Brain datasets are greater (>100 samples) than in other datasets. In these two datasets, *USP39* was distinctly upregulated in GBM compared with nonneoplastic brain tissue samples (Supplementary Fig. 1b).

**Fig. 1:**
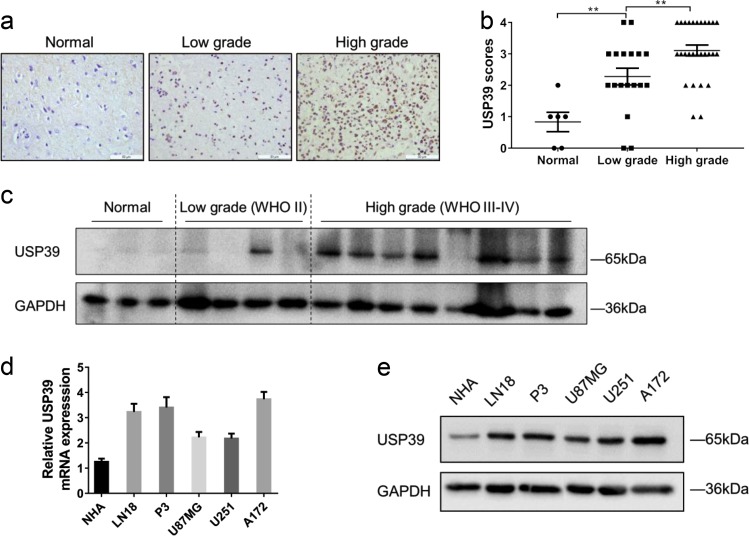
USP39 is overexpressed in glioma patient samples and glioma cells. **a** Representative images of IHC staining in human glioma and nonneoplastic brain tissue samples (*n* = 52). Scale bars, 50 µm. **b** Graphical representation of IHC scores in human glioma and nonneoplastic brain tissue samples. **c** Western blotting analysis for USP39 levels in lysates (20 µg) prepared from human glioma and nonneoplastic brain tissues (*n* = 15). **d** qRT-PCR and **e** western blotting analysis for USP39 levels in NHA, LN18, P3, U87MG, U251, and A172 cells. GAPDH was used for normalization. Student’s *t*-test: **p < 0.01

We validated these in silico results first through immunohistochemical (IHC) staining of primary human glioma samples. Staining for USP39 was increased in glioma samples (Fig. 1a), and levels in high-grade gliomas (WHO III–IV; *n* = 28) were increased compared with low-grade gliomas (WHO II; *n* = 18) and nonneoplastic human brain tissue samples (*n* = 6, Fig. 1b). These results indicated that USP39 protein expression was correlated with higher tumor grade. We verified that USP39 expression was associated with tumor grade, independent from other clinicopathological factors, including age, gender, tumor size, cystic change, and edema, which suggested that USP39 could be a potential diagnostic factor for glioma patients (Table 1, *p* < 0.05). Western blotting analysis of primary human glioma samples further supported these findings. USP39 protein levels were increased in high-grade glioma samples (WHO III–IV; *n* = 8) relative to low-grade glioma (WHO II; *n* = 4) and nonneoplastic human brain tissue samples (*n* = 3; Fig. 1c).

**Table 1:** Association between USP39 expression and clinicopathological factors in human glioma

Variables	No. of cases	USP39 expression		*p*-value
		Low	High	
Age (year)				
<60	32	10	22	0.4469
≥60	14	6	8	
Gender				
Male	29	9	20	0.4857
Female	17	7	10	
Tumor size (cm)				
<4	25	10	15	0.4176
≥4	21	6	15	
Cystic change				
Absent	18	7	11	0.6392
Present	28	9	19	
Edema				
None to mild	35	11	24	0.3942
Moderate to severe	11	5	6	
WHO grade				
Low grade (WHO II)	18	10	8	0.0177
High grade (WHO III–IV)	28	6	22	

We also examined USP39 mRNA and protein levels in several cell types in culture, including P3, LN18, U87MG, U251, and A172 glioma cells, and normal human astrocytes (NHA), using qRT-PCR and western blotting. USP39 mRNA and protein expression were significantly higher in the glioma cell populations than in NHA (1.9–4.2×; Fig. 1d, e). These data all together indicated that increased expression of USP39 is associated with increasing tumor grade in gliomas and that the protein may be involved in glioma progression.

### USP39 promotes proliferation, invasion, and migration of glioma cells in vitro

**Fig. 2:**
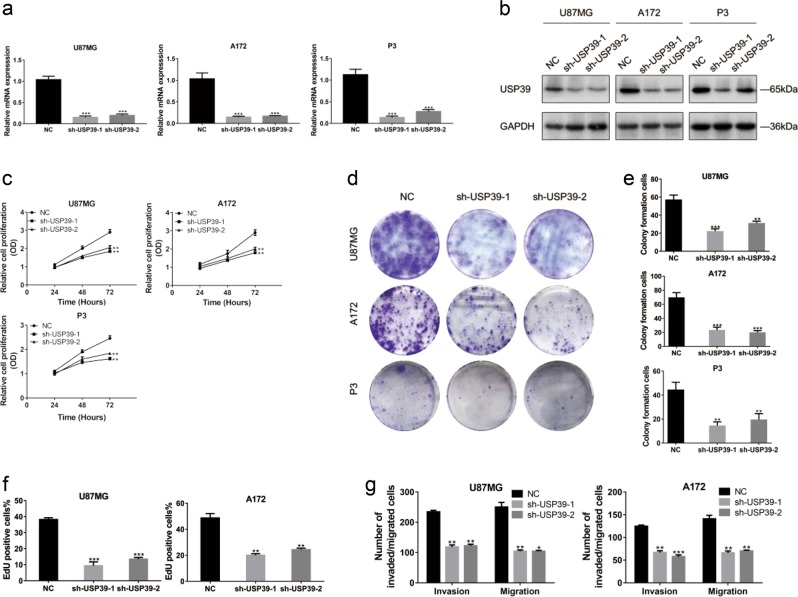
Knockdown of USP39 inhibits proliferation, invasion, and migration of glioma cells in vitro. **a** qRT-PCR analysis for USP39 mRNA levels in U87MG, A172, and P3 cells infected with two different USP39 shRNAs, sh-USP39-1 and -2. **b** Western blotting analysis of USP39 protein levels in U87MG, A172, and P3 cells infected with sh-USP39-1 and -2. **c** OD values from CCK8 assays of U87MG-, A172-, and P3-sh-USP39-1 and -2 plotted over time in hours to evaluate cell viability. **d**, **e** Representative images of colony forming assays for U87MG-, A172-, and P3-sh-USP39-1 and -2 to evaluate cell proliferation. Cells were fixed and stained with crystal violet. Colonies were counted, and results are represented in the bar graph. **f** Graphic representation of EdU-positive cells% in U87MG- and A172-NC, -sh-USP39-1, and -sh-USP39-2 cells. **g** Graphic representation of invaded and migrated cells counts from cell invasion and migration assays. Student’s *t*-test: **p < 0.01, ***p *<* 0.001

To examine the function of the protein in glioma development, we knocked down expression of USP39 in glioma cells in culture with short hairpin RNAs (shRNAs) in lentiviral constructs. Expression of USP39 was high in all glioma cells tested relative to NHA (Fig. 1e), so we randomly chose U87MG, A172, and P3 glioma cells for these experiments. ShRNAs targeting two different coding regions of USP39 (sh-USP39-1 and sh-USP39-2) were designed and tested in the three glioma cell populations. Both efficiently knocked down *USP39* mRNA by about 80% and protein expression by about 60% in all three glioma cell populations (Fig. 2a, b). Cell growth was significantly reduced in cells expressing the USP39 shRNAs as assessed in three different assays, including Cell Counting Kit-8 (CCK8), colony forming, and EdU assays (Fig. 2c–f and Supplementary Fig. 2a), compared with negative controls (NC). In cell invasion and migration assays, the number of invaded and migrated cells was reduced by nearly 50% in cells expressing the USP39 shRNAs (Fig. 2g and Supplementary Fig. 2b). These findings indicated that USP39 promoted proliferation, invasion, and migration of glioma cells in vitro.

### Downregulation of USP39 reduces cell growth in vivo

**Fig. 3:**
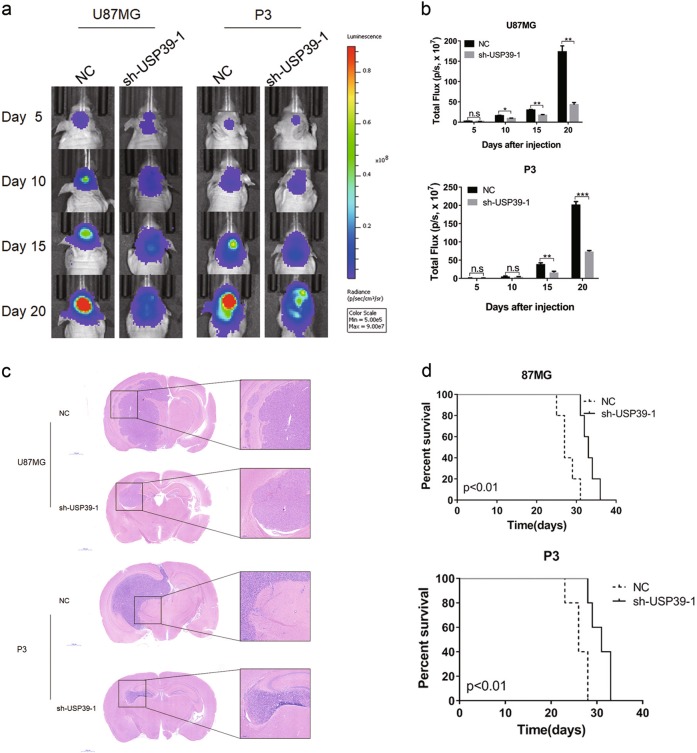
Knockdown of USP39 decreases proliferation, invasion, and migration of glioma cells in vivo. **a** Representative images of luciferase bioluminescence at the indicated days after injection of luciferase-expressing U87MG and P3 cells into the brains of nude mice. **b** Graphic representation of the total flux at the indicated days for the indicated cell types. **c** Representative images of HE staining of orthotopic xenografts derived from the indicated cell types, U87MG- and P3-sh-USP39-1 cells and controls. Scale bars, 1000 and 200 µm. **d** Kaplan–Meier analysis of survival for tumor bearing mice implanted with U87MG- and P3-sh-USP39-1 cells and controls. Student’s *t*-test: n.s. = not significant, *p *<* 0.05, **p < 0.01, ***p *<* 0.001. Log-rank test: *p* < 0.01

To examine the effect of USP39 on cell growth in vivo, we generated an orthotopic tumor model by implanting U87MG- and P3-NC/-sh-USP39-1 cells in the brains of nude mice. These cells were expressing luciferase, so that signal emanating from tumors could be measured over time at regular intervals. Bioluminescence signals did increase overall in mice over the 20-day time period (Fig. 3a), but USP39 knockdown led to a decreased rate in tumor growth. Total flux was used as a measure to quantify the in vivo bioluminescence imaging results. Total flux in U87MG-sh-USP39-1 tumors was markedly different at 10 days after injection of cells compared with U87MG-NC control tumors. Total flux was decreased by 45% at this time point. By 20 days, this difference had increased with values from U87MG-sh-USP39-1 tumors being 75% less than controls (Fig. 3b). The trend was the same for P3-sh-USP39-1 cells, although the reduced tumor growth of 58% relative to controls was not evident until 15 days after implantation (Fig. 3b).

Histological examination revealed that U87MG- and P3-sh-USP39-1 orthotopic xenografts tended to be less invasive and more circumscribed than controls (Fig. 3c). Furthermore, in Kaplan–Meier analysis of survival data from tumor bearing mice, animals implanted with U87MG- and P3-sh-USP39-1 cells exhibited better overall survival (median survival: 27 days vs. 33 days, U87MG-NC and U87MG-sh-USP39-1, respectively; 26 days vs. 31 days, P3-NC and P3-sh-USP39-1, respectively; Fig. 3d). Together, these findings demonstrated that USP39 depletion led to decreased cell growth in vivo.

### USP39 regulates TAZ independently of classical Hippo signaling

**Fig. 4:**
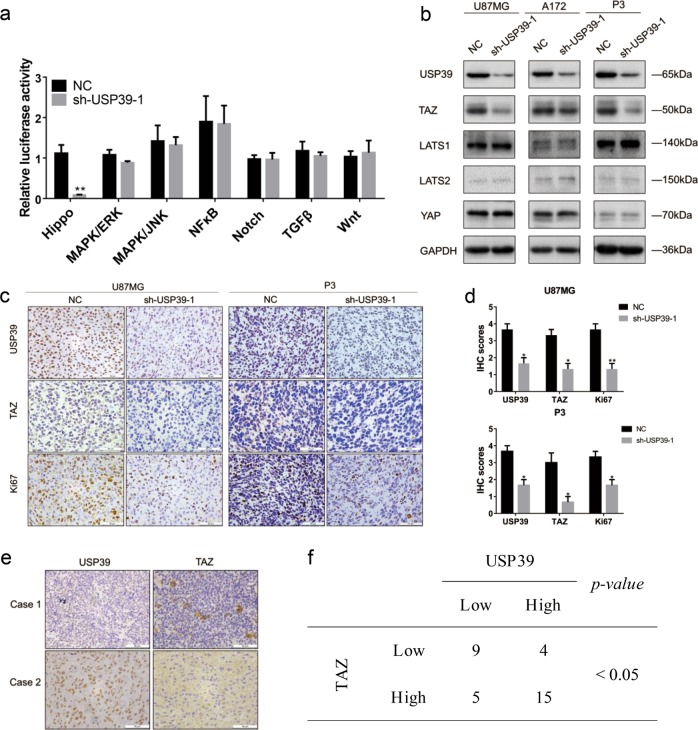
USP39 regulates TAZ through a LATS1/2-independent mechanism. **a** Graphic representation of relative luciferase activity in reporter assays to probe activation through seven cancer-associated pathways in U87MG-sh-USP39-1 cells. Luciferase reporter constructs regulated by pathway specific promoters were transfected into cells and assayed for luciferase activity. **b** Western blotting analysis of TAZ and other key components of the Hippo signaling pathway in U87MG-, A172-, and P3-sh-USP39-1 cells relative to controls. GAPDH was used for normalization. **c** Representative images of IHC staining of USP39, TAZ, and Ki67 levels in xenograft sections from U87MG-NC, U87MG-sh-USP39-1, P3-NC, and P3-sh-USP39-1 groups. Scale bars, 50 µm. **d** Graphic representation of IHC scores for USP39, TAZ, and Ki67 levels from indicated groups. **e** Representative images of IHC staining of USP39 and TAZ in primary human glioma samples (*n* = 33). Scale bars, 50 µm. **f** Correlation of USP39 and TAZ protein expression in primary human glioma samples. IHC scores are indicated in parentheses. Student’s *t*-test: *p < 0.05, **p < 0.01. χ^2^-test and Fisher’s exact test: *p* < 0.05

To explore the potential mechanisms underlying USP39-induced malignant behaviors in glioma, we transfected a series of luciferase reporter constructs to assay signaling activity from seven different pathways, such as Notch, Hippo, and Wnt, which are typically dysregulated in human cancers. In these constructs, luciferase is regulated by a transcriptional element specific to each pathway. We found that the luciferase activity from a synthetic TEAD-dependent YAP/TAZ-responsive reporter, as a direct readout of Hippo activity, was decreased by 92% in U87MG-sh-USP39-1 cells, whereas the luciferase activities of the other six cancer pathways, including MAPK/ERK, MAPK/JNK, NFκB, Notch, TGFβ, and Wnt, remained unchanged (Fig. 4a).

Western blotting analysis showed that TAZ protein expression was also reduced in U87MG-, A172-, and P3-sh-USP39-1 cells relative to controls, but other key components of the Hippo pathway, including LATS1, LATS2, and YAP, remained unchanged (Fig. 4b). In view of the critical role of YAP/TAZ in activating transcription and YAP protein remained unchanged in our work, we validate the individual effect of TAZ using luciferase assays (Supplementary Fig. 3a). We hypothesized that USP39 might promote malignant behaviors through TAZ. We performed IHC on sections from the xenografts derived from the modified cell types to determine the relationship between these proteins in vivo. The IHC scores demonstrated that TAZ protein expression was decreased after knockdown of USP39 (Fig. 4c, d). In addition, in line with the results from Fig. 3, IHC scores for Ki67, a marker for proliferation, were correspondingly decreased (Fig. 4c, d). Finally, we performed IHC on primary human glioma samples (*n* = 33) and found that IHC scores for USP39 correlated with scores obtained for TAZ (*p* < 0.05; Fig. 4e, f). Thus, USP39 and TAZ proteins were positively correlated. All together, these results demonstrated that loss of USP39 led to decreased TAZ but not other components of the Hippo signaling pathway including LATS1/2.

### USP39 regulates TAZ protein expression through *TAZ* mRNA maturation

**Fig. 5:**
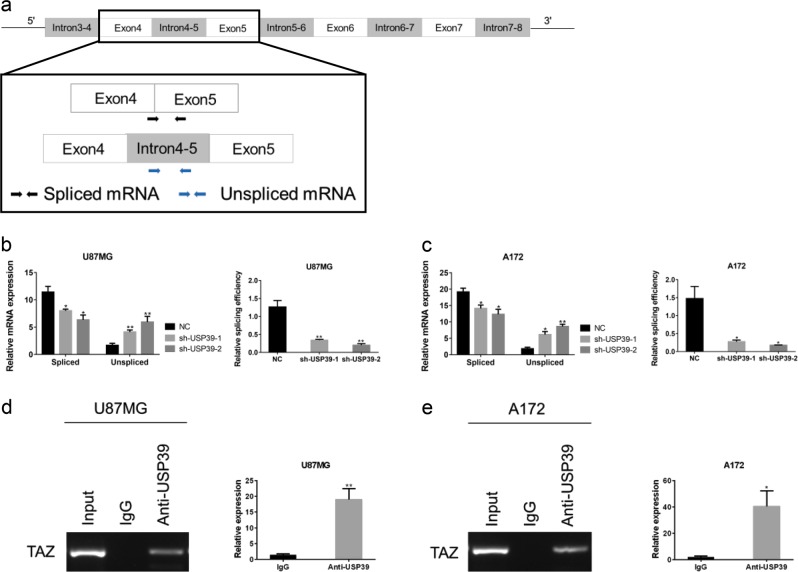
Knockdown of USP39 decreases TAZ protein levels by reducing the splicing of *TAZ* pre-mRNA. **a** Schematic model (top) representing the region common among the three longest primary transcripts of *TAZ* predicted from the Ensembl database. Graphic representation (bottom) of the region from exon 4 to exon 5 selected for amplification experiments to detect spliced and unspliced RNA transcripts using qRT-PCR. Specific primers designed are represented by the two arrow pairs, which illustrate their approximate locations. **b**, **c** qRT-PCR analysis showing relative mRNA levels of spliced and unspliced *TAZ* RNA transcripts and splicing efficiency in U87MG- and A172-sh-USP39-1 and -2 cells relative to controls. GAPDH was used for normalization. **d**, **e** RNA protein immunoprecipitation was performed using anti-USP39 (with IgG as control), and qRT-PCR were used to detect the level of *TAZ* mRNA in the immunoprecipitated complex. Student’s *t*-test: *p < 0.05, **p < 0.01

USP39 has been reported to be involved in pre-mRNA splicing of certain genes [6–8, 35]. We therefore investigated whether USP39 had a role in posttranscriptional regulation of *TAZ* mRNA splicing. Little is currently known about mRNA splicing of TAZ transcripts and their final form. However, according to the Ensembl database, TAZ has three predicted primary transcripts, which could yield a translated product corresponding to the longest protein isoform of TAZ observed in this work. Although these predicted primary transcripts have different lengths, they share some common regions. For example, in the longest primary transcript, the region from introns 3–4 to introns 7–8 is also contained in two other primary transcripts. Because these exons (exons 4–7) are equally represented in the different transcripts, the region from exons 4 to 5 in the longest primary transcript was selected to design specific primers to distinguish *TAZ* spliced mRNA and *TAZ* unspliced mRNA (Fig. 5a). Using qRT-PCR, we detected increased levels of unspliced mRNA transcripts in U87MG-sh-USP39-1 and A172-sh-USP39-1 cells relative to controls, whereas spliced mRNA was significantly decreased (~70%; Fig. 5b, c). Besides, we performed the RNA-binding protein immunoprecipitation assay (RIP) on extracts prepared from U87MG and A172 using USP39 antibody. The results of qRT-PCR demonstrated that TAZ expression was significantly enriched in USP39 pull downs compared with IgG controls (Fig. 5d, e). These data suggested that knockdown of USP39 led to decreased TAZ protein levels at least in part by suppressing splicing of its pre-mRNA.

### Downregulation of USP39 decreases TAZ transcriptional activity

**Fig. 6:**
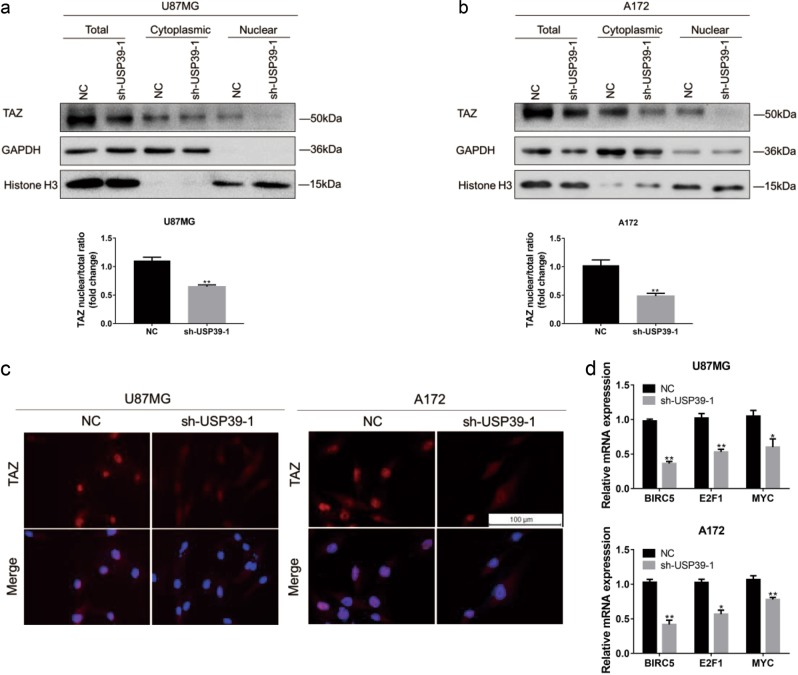
Downregulation of USP39 decreases TAZ transcriptional activity. **a**, **b** Western blotting analysis of total, cytoplasmic, and nuclear fractions prepared from indicated cells. Bar graph showing the ratio of TAZ nuclear protein (normalized to Histone H3) to total protein (normalized to GAPDH) levels in modified and control (NC) cells. **c** Representative images of immunofluorescence for TAZ (red) in indicated cells. Nuclei are stained with DAPI (blue). Scale bars, 100 µm. **d** qRT-PCR analysis for expression of TAZ target genes in U87MG- or A172-sh-USP39-1 compared with controls. GAPDH was used for normalization. Student’s *t*-test: n.s. = not significant, *p < 0.05, **p < 0.01, ***p < 0.001

To better clarify the regulation of TAZ by USP39, cellular localization of the TAZ protein was examined. Knockdown of USP39 was performed in U87MG and A172 cells, and lysates were prepared from nuclear and cytoplasmic fractions. In western blotting analysis, we observed a strong decrease in the ratio of TAZ nuclear to total protein levels in two cell populations with USP39 loss relative to controls (Fig. 6a, b). Immunofluorescence staining was consistent with these findings (Fig. 6c). Finally, transcriptional activity paralleled the loss of nuclear TAZ; mRNA levels of TAZ target genes, *BIRC5*, *E2F1*, and *MYC* were decreased (Fig. 6d) [22, 24, 36]. These data therefore indicated that USP39 was involved in the transcriptional activation of TAZ.

### Ectopic expression of USP39 and TAZ restores malignant properties of glioma cells with the loss of USP39 in vitro and in vivo

**Fig. 7:**
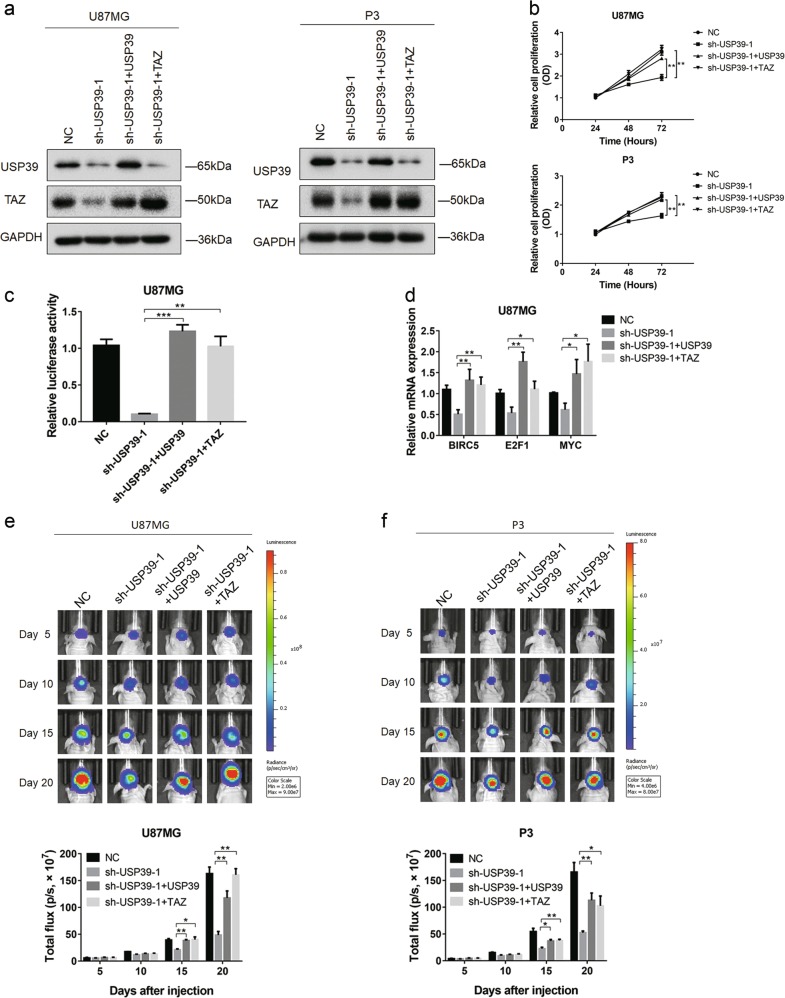
Ectopic expression of USP39 and TAZ restores malignant properties of glioma cells with knockdown of USP39 in vitro and in vivo. **a** Western blotting analysis of lysates prepared from modified U87MG and P3 cells. GAPDH was used for normalization. **b** OD from CCK8 assays plotted as a function of time in hours for indicated cells to evaluate cell viability. **c** Graphic representation of relative luciferase activity from luciferase reporter constructs regulated by Hippo signaling in U87MG cells from indicated groups. **d** qRT-PCR analysis of the expression of TAZ target genes from indicated cells. GAPDH was used for normalization. **e**, **f** Representative images of luciferase bioluminescence at the indicated days after injection of U87MG and P3 cells into the brains of nude mice. Graphic representation of the total flux at the indicated days for the indicated cell types. Student’s *t*-test: *p < 0.05, **p < 0.01, ***p < 0.001

To further validate the putative oncogenic role of USP39 in glioma and exclude off-target effects of the shRNAs, USP39 was overexpressed in cells with depletion of USP39. To investigate the functional role of TAZ protein in the USP39-promoted pathway in glioma, U87MG-, and P3-sh-USP39-1 cells were infected with a wild-type TAZ lentiviral expression construct. Efficiency of expression from these constructs was assessed by western blotting; USP39 or TAZ was increased in both U87MG- and P3-sh-USP39-1 cells (Fig. 7a).

Ectopic expression of USP39 or TAZ in U87MG- and P3-sh-USP39-1 cells restored malignant properties. Cell proliferation reached levels observed in controls without the loss of USP39 (Fig. 7b). In addition, luciferase activity, from a Hippo pathway regulated luciferase reporter construct, was rescued in U87MG-sh-USP39-1 cells as well as transcription of genes downstream of TAZ (Fig. 7c, d). These in vitro results were further validated in vivo through orthotopic implantation of the various modified cell types. Cell growth in U87MG- and P3-sh-USP39-1 cells with USP39 or TAZ overexpression reached values similar to those obtained from control cells (Fig. 7e, f). These data overall demonstrated that TAZ was a critical downstream effector mediating the oncogenic function of USP39 in glioma.

## Discussion

Previous studies have reported that USP39 contributes to cancer progression and predicts poor prognosis in various tumors [10–21]. For example, USP39 has been shown to be essential for KRAS-driven cancer [35]. Here, analysis of expression data from the Oncomine dataset demonstrated that *USP39* mRNA might be upregulated in GBM. We found increased protein levels of USP39 to be associated with higher tumor grade in an independent cohort of primary human gliomas. In functional experiments, we demonstrated that this increase in USP39 promotes cell proliferation, invasion, and migration in vitro and in vivo. These results are consistent with an oncogenic role for USP39 in the development of human glioma.

Based on a series of luciferase reporter assays designed to probe activity in seven cancer-associated pathways, we found that events downstream of Hippo signaling were dramatically affected by USP39 levels in glioma. Previous work from our lab supports a critical role for Hippo signaling in the development of human glioma. We found that actin like-6A (ACTL6A) promotes glioma progression by directly associating with YAP/TAZ, which prevents ubiquitination of YAP/TAZ protein. We also observed that ACTL6A mainly interacts with the C-terminal region of YAP, thus inhibiting YAP from binding to the ubiquitin ligase β-TrCP [37]. Other studies have also demonstrated a critical role for YAP/TAZ in gliomagenesis [33, 34, 38]. Therefore, we aim to identify novel mechanisms of regulation for Hippo signaling in human gliomas.

We carefully investigated the relationship between USP39 and Hippo signaling through protein analysis of key components of the pathway. Protein levels of TAZ were reduced with USP39 knockdown, but other key components in the Hippo pathway, including LATS1/2 and YAP, remained unchanged. The existence of a potential link between the protein expression of USP39 and TAZ was also supported by IHC performed on sections from xenografts derived from modified U87MG and P3 cells, as well as human primary glioma samples. Although YAP and TAZ have been reported to act synergistically to activate transcription [39], overexpression of TAZ or knockdown of TAZ alone has been found to substantially affect Hippo signaling (8 × GTIIC-Luc reporter) [40]. We verified this result in glioma, which explains why Hippo activity could be impaired even in the presence of normal protein levels of YAP. The decrease in Hippo signaling (~90%) also did not parallel the reduction in TAZ protein levels (~50%) after USP39 knockdown. TAZ is a transcriptional coactivator, which cannot directly bind to DNA, but it interacts with several oncogenic transcription factors, including TEADs, to activate transcription. A study has reported that TAZ mutants defective in interaction with TEADs failed to accumulate in the nucleus [41]. So one plausible interpretation would be that USP39 may also affect the interaction between TAZ and transcription factors, which might contribute to inhibition of Hippo signaling.

A fundamental question that remains is how USP39 drives the increase in TAZ protein. USP39 is reported to be a splicing factor and to take part in the processing of the mRNA of some genes [6–8, 35]. Although the regulation of TAZ expression has been thoroughly investigated at the transcriptional level [42], the mechanisms governing the processing of *TAZ* mRNA are less well understood. We provide evidence that USP39 regulates TAZ protein levels, at least in part, by controlling mRNA maturation. According to the Ensembl database, there are three primary transcripts that can be translated into full-length TAZ protein. For detection of unspliced and spliced *TAZ* mRNAs, we designed two primer pairs specific for a region common to all three primary transcripts. This method has been previously used to determine the splicing rate of various gene transcripts [10, 43]. Indeed, a decrease in *TAZ* pre-mRNA splicing efficiency was detected in cells with USP39 knockdown, implicating a role for the protein in the maturation of *TAZ* mRNA. Furthermore, USP39 plays a central role in pre-mRNA splicing of many genes, including *CASP8AP2*, *CDCA8*, *MED19*, *SERPINB6*, *TAF9B*, *BORA*, *KIF14*, and *ORC11* [35]. Combined with results from previous studies [5, 9, 10], it is reasonable to speculate that the effects of USP39 on the maturation of mRNA are not merely specific to *TAZ* mRNA.

We also demonstrated that USP39 increases TAZ protein level and thus enhances its nuclear function. However, the mechanism linking USP39 to TAZ activation is unclear. TAZ has been identified as an integral component of the β-catenin destruction complex, which effectively sequesters TAZ in the cytoplasm, and knockdown of USP39 has also been shown to lead to decreased protein expression of β-catenin [44–46]. USP39 silencing might therefore enhance the β-catenin destruction complex, and thus lead to cytoplasmic retention and reduced nuclear localization of TAZ. Further investigation is necessary to illuminate the specific mechanism.

In summary, our work indicated that upregulated expression of USP39 is a common event in human gliomas, and that it promotes malignant tumor properties in gliomas both in vitro and in vivo. Thus, USP39 appears to have oncogenic properties in the development of human gliomas. Our study further demonstrated that the oncogenic activity of USP39 is due to its ability to activate TAZ. However, this activity is independent of canonical Hippo signaling, and is instead due to its ability to enhance pre-mRNA splicing of *TAZ*. Finally, our work provides an important basis for the development of diagnostic and therapeutic approaches in the treatment of glioma patients using USP39.

## Materials and methods

### Cell lines and cell culture

Human glioma cell lines LN18, U87MG, A172, and U251 were obtained from the Culture Collection of the Chinese Academy of Sciences (Shanghai, China). NHA and primary GBM#P3 cells were kindly provided by Professor Rolf Bjerkvig (University of Bergen; Bergen, Norway). P3 cells were cultured in Dulbecco’s modified Eagles’s medium (DMEM)/F-12 medium (Thermo Fisher Scientific; Waltham, MA, USA) supplemented with 2% B27 Neuro Mix (Thermo Fisher Scientific), 20 ng/mL epidermal growth factor (EGF; Thermo Fisher Scientific), and 10 ng/mL basic fibroblast growth factor (βFGF; PeproTech; Rocky Hill, NJ, USA). All other cells were cultured in DMEM (Thermo Fisher Scientific) supplemented with 10% fetal bovine serum (FBS; Thermo Fisher Scientific). Cells were maintained at 37 °C in a humidified chamber containing 5% CO_2_.

### Lentiviral transductions

U87MG, A172, or P3 cells were infected with lentivirus containing two different shRNAs targeting USP39 (sh-USP39-1, sh-USP39-2; OBiO Technology, Shanghai, China). After 48 h, cells were cultured in DMEM or DMEM/F-12 containing puromycin (2 μg/mL; Thermo Fisher Scientfic) for an additional 2 weeks to select for cells containing the constructs. For ectopic expression of USP39 or TAZ, U87MG- and P3-sh-USP39-1 cells were infected with lentiviral constructs expressing full-length USP39 or TAZ (OBiO Technology). The sequences of the shRNAs and siRNAs used are the following: sh-negative control, 5′-UUCUCCGAACGUGUCACGUTT-3′; sh-USP39-1, 5′-GCAUAUGAUGGUACCACUUTT-3′; sh-USP39-2, 5′-CCUUCAGGCUCUAUCUAAUTT-3′; si-negative control, 5′-UUCUCCGAACGUGUCACGUTT-3′; si-YAP, 5′-GACTCAGGATGGAGAAATTTA-3′; si-TAZ, 5′-GCTCATGAGTATGCCCAAT-3′.

### Nuclear fractionation

Nuclear and cytoplasmic fractions from different cell populations were isolated using Nuclear and Cytoplasmic Extraction Reagents (Thermo Fisher Scientific), according to the manufacturer’s instructions. GAPDH and Histone H3 were used for normalization.

### Immunohistochemistry

Glioma samples were obtained from 46 patients (WHO II–IV) who had undergone surgeries performed at the Department of Neurosurgery at the Qilu Hospital. Nonneoplastic brain tissue samples were collected from six patients who underwent partial resection due to trauma or other conditions unrelated to cancer. Tissues were fixed with 4% formalin, embedded in paraffin, and sectioned (4 µm). IHC assays were performed using the SPlink Detection Kit and DAB (ZSGB-BIO; Beijing, China) according to the manufacturer’s instructions. The following primary antibodies were used: USP39 (#ab131332, 1:200, Abcam, Cambridge, MA, USA), TAZ (#ab84927, 1:200, Abcam), and Ki67 (#ab92742, 1:800, Abcam). USP39 staining was scored as follows: 0, no staining; 1, weak staining in <50% cells; 2, weak staining in ≥50% cells; 3, strong staining in <50% cells; and 4, strong staining in ≥50% cells.

### Immunofluorescence

Cells were cultured in 24-well plates, fixed with 4% paraformaldehyde, permeabilized with 0.4%Triton X-100, blocked with 5% bovine serum albumin, and incubated with primary antibody against TAZ (#8418, 1:200, Cell Signaling Technology, Beverly, MA, USA) at 4 °C overnight. Primary antibody was detected with an Alexa Fluor 594 conjugated goat anti-rabbit IgG antibody (#ab150080, 1:800; Abcam), and cell nuclei were stained with DAPI (Sigma-Aldrich; St. Louis, MO, USA). Images were obtained under fluorescence microscopy (Leica; Wetzlar, Germany).

### Western blotting analysis

Cells and tissues were lysed in RIPA Lysis and Extraction Buffer (Thermo Fisher Scientific) containing a protease inhibitor cocktail (Sigma-Aldrich). Protein lysates (20 μg) were run on SDS-PAGE and transferred onto polyvinylidene difluoride membranes. Membranes were blocked in 5% skim milk, and incubated overnight with primary antibodies at 4 °C followed by incubation with secondary antibodies (ZSGB-BIO). Proteins on membranes were visualized using the Chemiluminescent Reagents Kit (Millipore; Billerica, MA, USA). Signals were detected with the Chemi-Doc XRS+ (Bio-Rad; Hercules, CA, USA) and quantified using Image Lab 3.0 software (Bio-Rad). The following primary antibodies were used for western blotting: USP39 (#ab131332, Abcam), Histone H3(#ab176842, Abcam), Ki67 (#ab92742, Abcam), GAPDH (#sc-25778, Santa Cruz Biotechnology; Dallas, TX, USA), LATS1(#3477, Cell Signaling Technology), LATS2(#5888, Cell Signaling Technology), YAP (#14074, Cell Signaling Technology), and TAZ (#8418, Cell Signaling Technology).

### Cell invasion and migration assay

Cells (2 × 10^4^/well) were seeded into the upper chamber of transwell permable supports (pore size: 8 μm; Corning Costar; Tewksbury, MA, USA), and medium containing 30% FBS (600 μL) was added into the lower chamber. For invasion assays, the upper chamber was coated with Matrigel (BD Biosciences; Bedford, MA, USA). Chambers were incubated at 37 °C for 24–36 h, and cells were fixed with 4% paraformaldehyde and stained with crystal violet (Solarbio; Beijing, China). Images were obtained from five random fields (×100) in each well. All experiments were performed in triplicate.

### Cell viability assay

Cells (5 × 10^3^/well) were seeded into 96-well plates and incubated overnight. CCK8 solution (Dojindo; Kumamoto, Japan) was added to evaluate cell viability every 24 h according to the manufacturer’s instructions. Samples were measured at 450 nm in a microplate reader (PerkinElmer; San Jose, CA, USA).

### EdU assay

Cells (2 × 10^4^/well) were seeded into 24-well plates and incubated overnight. Proliferation was evaluated using the EdU Kit (Rib-bio; Guangzhou, China) according to the manufacturer’s instructions. Images were obtained from three random fields (×200) under fluorescence microscopy (Leica).

### Colony forming assay

U87MG and A172 cells (1 × 10^3^/well) were seeded into 6-well plates, and P3 cells were seeded into 6-well plates coated with poly-l-lysine (Solarbio). After 2 weeks, cells were fixed with 4% paraformaldehyde and stained with crystal violet (Solarbio). The total number of colonies was counted per well. All experiments were performed in triplicate.

### Reverse transcription PCR

Cell and tissue RNA isolation were carried out using TRIzol Reagent (Takara; Tokyo, Japan) according to manufacturer’s instructions. Isolated total RNA was quantified and used to generate cDNA using a reverse transcription kit (Toyobo; Osaka, Japan). mRNA levels were quantified by real-time PCR. GAPDH served as an internal control. The following primers were used:

*GAPDH*, forward, 5′-GCACCGTCAAGGCTGAGAAC-3′, reverse, 5′-TGGTGAAGACGCCAGTGGA-3′; *USP39*, forward, 5′-TGACCTCATTGCCAACATCGT-3′, reverse, 5′-TTGCCTGTCCCATGATGAAGC-3′; *BIRC5*, forward, 5′-TGACGACCCCATAGAGGAACA-3′, reverse, 5′-CGCACTTTCTCCGCAGTTTC-3′; *E2F1*, forward, 5′-GCCATCCAGGAAAAGGTGTGA-3′, reverse, 5′-GTGATGTCAGATGCGCCG-3′; *MYC*, forward, 5′-GGTAGTGGAAAACCAGCAGCC-3′, reverse, 5′-TTCTCCTCCTCGTCGCAGTA-3′; *TAZ* unspliced mRNA, forward, 5′-GGCTGCAATACCGTGTTACC-3′, reverse, 5′-CAAAGTCACCCAAAGCGTGT-3′; *TAZ* spliced mRNA, forward, 5′-CCCAGCCAAATCTCGTGATGAA-3′, reverse, 5′-AGCGCATTGGGCATACTCAT-3′; *TAZ* mRNA, forward, 5′-GACTCTTGCCCCAGTTCAGG-3′, reverse, 5′-CCATTGAGGAAAGGATCTGAGC-3′.

### Luciferase reporter assays

Cells were seeded in 96-well plates (3 × 10^3^/well). After 24 h, luciferase reporter constructs containing a transcription factor responsive element upstream of firefly luciferase were co-transfected with a Renilla luciferase construct into cells using Lipofectamine 3000 (Thermo Fisher Scientific), according to the manufacturer’s instructions. After 24 h, luciferase activity was analyzed using the Dual Luciferase Reporter Assay kit (Promega) according to the manufacturer’s protocol. Renilla activity was used to normalize luciferase reporter activity. Experiments were performed in triplicate. Plasmids used were the following: firefly luciferase driven by Elk-1/SRF, AP-1, NFκB, RBP-Jκ, SMAD2/SMAD3/SMAD4, or TCF/LEF (Qiagen; Hilden, Germany) was co-transfected with Constitutively expressing Renilla luciferase construct (Qiagen); YAP/TAZ-responsive luciferase reporter construct, 8 × GTIIC-Luc (Addgene; Cambridge, MA, USA) was co-transfected with pGL4-SV40 driven Renilla luciferase (Promega; Madison, WI, USA).

### RNA-binding protein immunoprecipitation assay

RIP analysis was performed using the Magna RIP RNA-binding protein immunoprecipitation kit (Millipore, Billerica, MA, USA) and the USP39 antibody (#ab131332, Abcam) following the manufacturer’s protocol. Co-precipitated RNAs were isolated, purified, and subjected to qRT-PCR analysis.

### Animal studies

Athymic BALB/c nude mice (male, 3- to 4-week-old; GemPharmatech Co., Ltd; Nanjing, China) were randomly divided into indicated groups. Luciferase-expressing cells (1 × 10^6^) were inoculated into the frontal lobe using a stereotactic apparatus (KDS310, KD Scientific; Holliston, MA, USA). Bioluminescence images were captured using an imaging system (PerkinElmer) every 5 days. When animals were displaying symptoms, such as apathy, decreased activity, severe hunchback posture, dragging legs, unkempt fur, or loss of body weight, they were killed by cervical dislocation. If necessary, animals were perfused with saline solution and 4% paraformaldehyde, and excised brains were prepared for further examination by HE and IHC staining.

### Statistical analysis

All experiments were carried out with at least three replicates. Data were expressed as the mean ± SEM, and statistical significance was calculated with unpaired two-tailed, Student’s *t*-tests, unless otherwise stated. Survival curves were estimated by the Kaplan–Meier method and compared using the log-rank test. A two-sided χ^2^-test and Fisher’s exact test were both used to determine the association between USP39 and TAZ. A two-sided χ^2^-test was used to determine the association between USP39 expression and clinicopathological factors. GraphPad Prism version 7.00 software program (GraphPad; La Jolla, CA, USA) was used to analyze data from in vitro and in vivo experiments. Differences were considered as statistically significant when *p* < 0.05.

## Supplementary information

Supplemental information

Supplemental figure 1

Supplemental figure 2

Supplemental figure 3
