# Supplementary material for: RNA splicing factor USP39 promotes glioma progression by inducing TAZ mRNA maturation
Source: Oncogene. 2019 Jul 22;38(37):6414–28. doi: 10.1038/s41388-019-0888-1 (PMC6756117; doi:10.1038/s41388-019-0888-1)
Supplement: Supplementary file 1 — Supplemental information [file 41388_2019_888_MOESM1_ESM.doc]

**Supplemental Figure 1**

(a) Analysis of USP39 mRNA expression profiles across multiple cancer types compared to normal tissues using the publicly available Oncomine datasets. The number in the colored cell represents the number of analyses meeting thresholds. Cell color was determined by the gene rank. The more intense red (over-expression) or blue (under-expression) colors correspond to a more highly significant over-expressed or under-expressed gene. (b) Oncomine data mining for USP39 mRNA levels in TCGA Brain, Sun Brain, Murat Brain, Liang Brain, Lee Brain and Bredel Brain datasets between normal tissues versus glioblastoma.

**Supplemental Figure 2**

(a) EdU assay for U87MG- and A172-NC, -sh-USP39-1, and -sh-USP39-2 cells. Nuclei are stained with Hoechst33324 (blue). Scale bars, 100 µm. (b) Representative images of invasion and migration assays for U87MG- and A172-sh-USP39-1 and -2 cells and controls to evaluate cell invasion and migration. Cells were fixed and stained with crystal violet. Scale bars, 200 µm.

**Supplemental Figure 3**

1. Luciferase assay for 8×GTIIC-Lux or control reporter indicating YAP/TAZ dependent transcriptional activity in U87MG cells transfected with siRNAs as indicated. Data are normalized to a Renilla reporter and to negative control (NC). Student’s *t*-test: ***p* < 0.01, ****p* < 0.001
