# Supplementary figures and images for: RNA splicing factor USP39 promotes glioma progression by inducing TAZ mRNA maturation
Source: Oncogene. 2019 Jul 22;38(37):6414–28. doi: 10.1038/s41388-019-0888-1 (PMC6756117; doi:10.1038/s41388-019-0888-1)

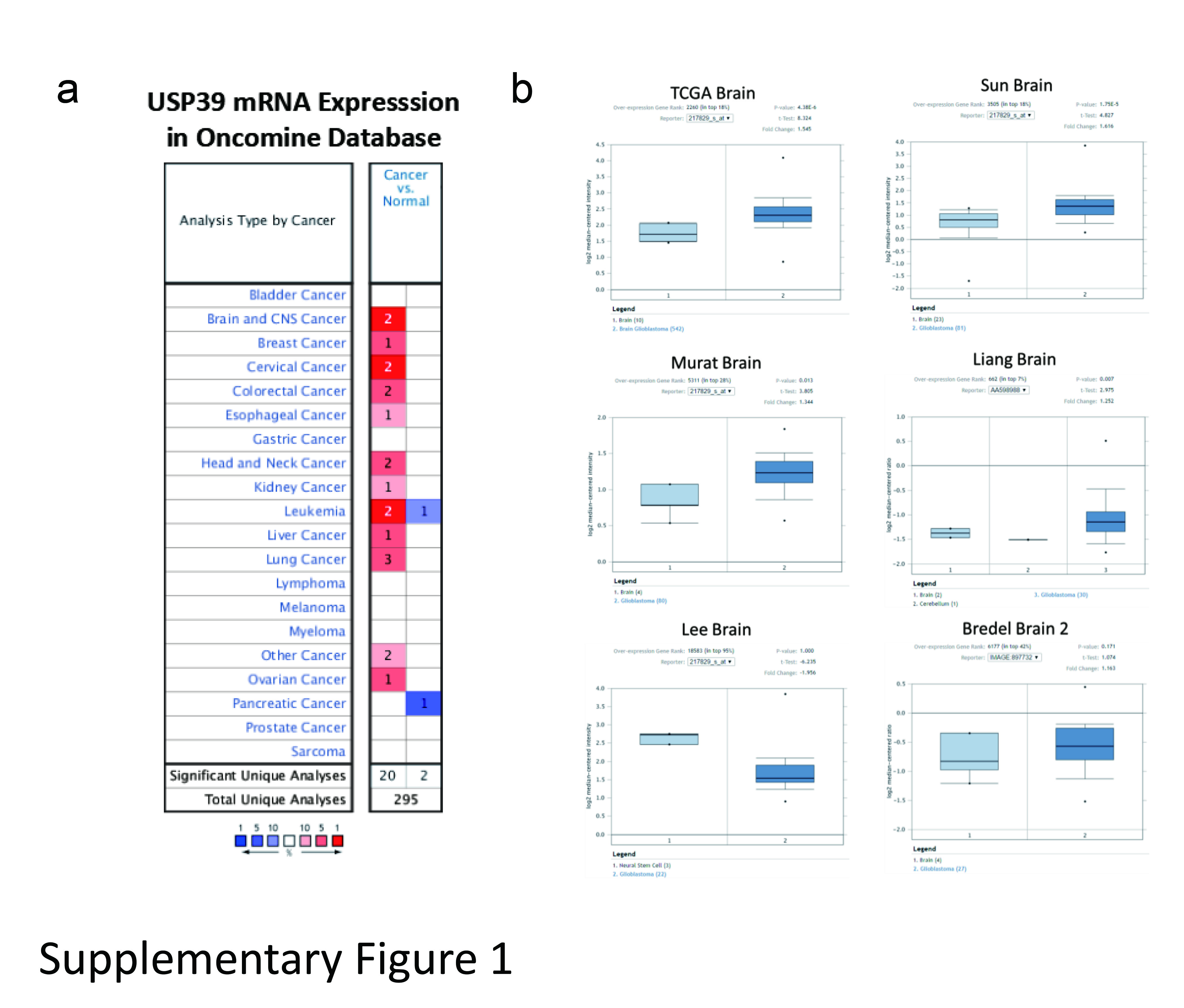

Supplement: Supplementary file 2 — Supplemental figure 1 [file 41388_2019_888_MOESM2_ESM.jpg]

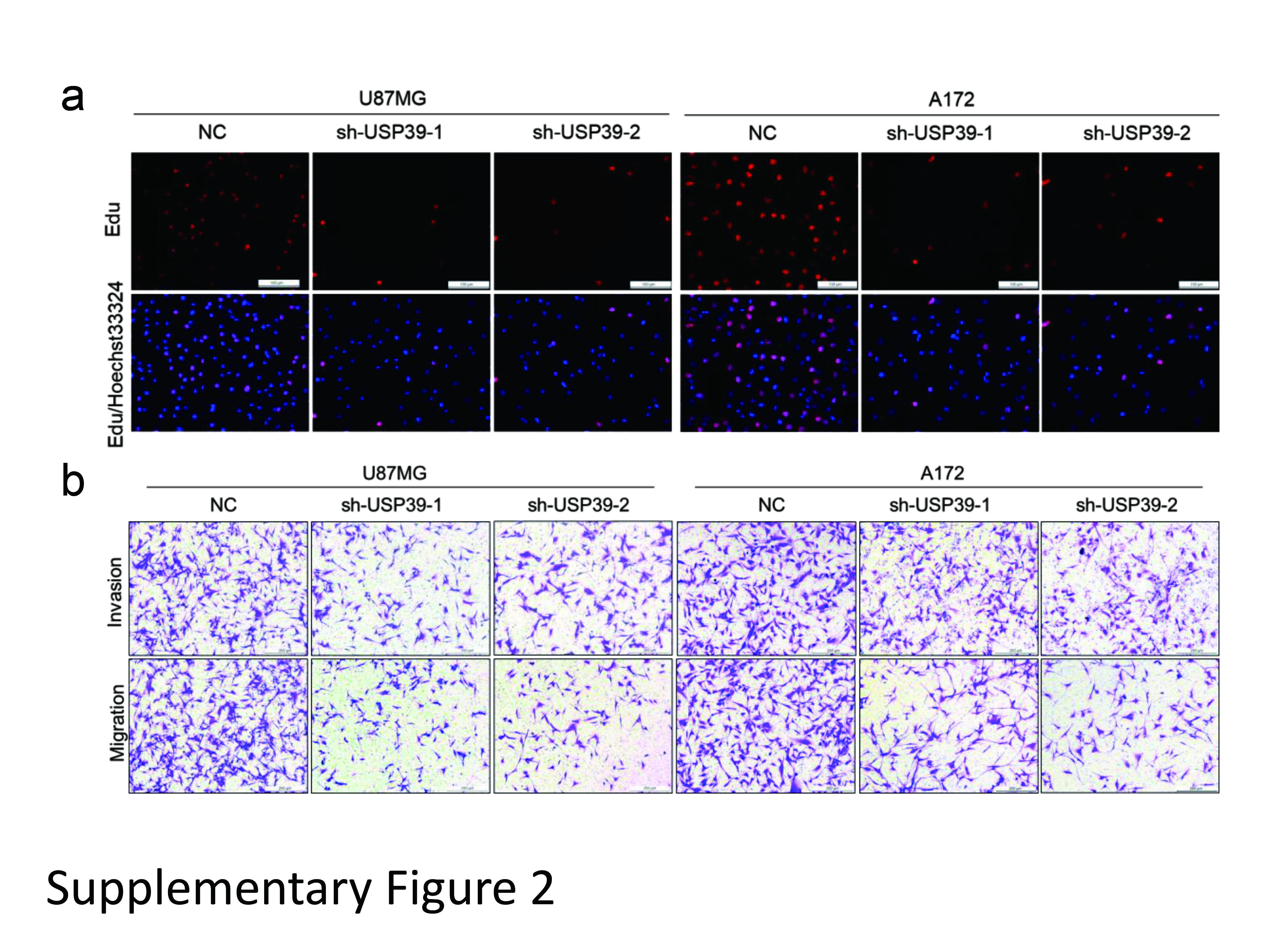

Supplement: Supplementary file 3 — Supplemental figure 2 [file 41388_2019_888_MOESM3_ESM.jpg]

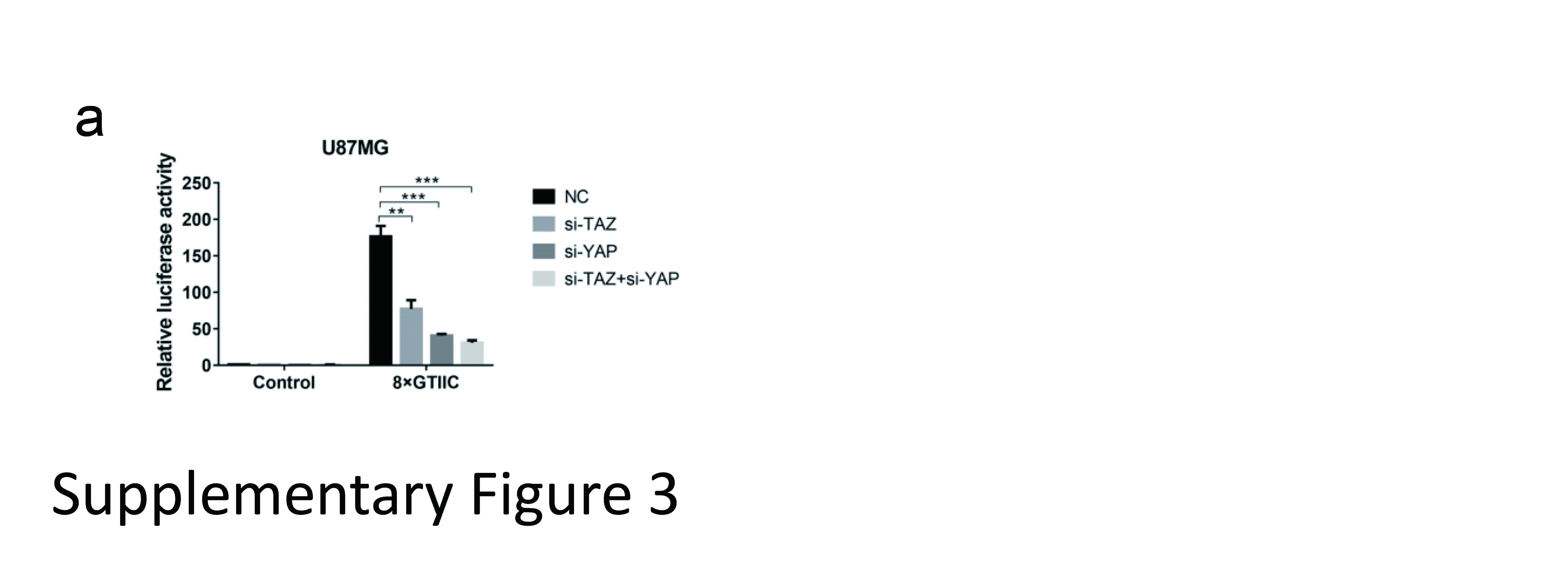

Supplement: Supplementary file 4 — Supplemental figure 3 [file 41388_2019_888_MOESM4_ESM.jpg]
